# A regional look at ethics in healthcare

**DOI:** 10.4102/phcfm.v11i1.2172

**Published:** 2019-10-09

**Authors:** Anita Kleinsmidt

**Affiliations:** 1 Centre for Medical Ethics and Law, Faculty of Medicine and Health Sciences, Stellenbosch University, Cape Town, South Africa

This book is volume 13 of the ‘Advancing Global Bioethics’ series. The series aims to provide a forum for analysis of issues in bioethics from global and cross-cultural perspectives. The issues covered by the series include scientific misconduct, brain drain and migration of healthcare workers, indigenous medicine, malnutrition, human rights and climate change.

This book in the ‘Advancing Global Bioethics’ series, looks at medical ethics through the lens of African traditional customs and beliefs from the perspectives of different categories of health professionals. The book is structured as follows: the first 3 chapters introduce and describe the development of bioethics in Africa, followed by bioethics and professionalism in the healthcare context and the ubuntu-botho approach to ethics.

The remaining 15 chapters are divided by health profession into chapters highlighting bioethics in occupational therapy, nutrition and dietetics, social work, physiotherapy, nursing, radiography and finally public health. Each discipline has a chapter with an African perspective, followed by a chapter on the same discipline from a South African perspective. This makes for interesting comparison of the ethical issues highlighted within a profession from both African and South African points of view.

Western bioethics in the principalist and Kantian traditions has traditionally been focussed on individual rights, and this book highlights a critique from Africans that not enough emphasis has been placed on solidarity and justice. This book promotes a system-focussed approach ‘that is responsive to the demographics, culture and community contexts of patients’ rather than applying a western ethical analysis to the African context.

The ‘Ubunto-botho approach to ethics’ chapter critiques the western approach adopted by psychologists, that is, ‘an objective disinterested stance towards the object of one’s knowledge’. Kantian ethics is also mentioned as running counter to indigenous African epistemologies. The relationship between ethics and culture is discussed. Causes of illness, autonomy, treatment – not just of the individual but of the community – are not necessarily perceived in a purely biological sense but include an ethno-biological view of a dynamic relationship between the individual and the clan, which includes relationships with deceased ancestors and those unborn. This is important for understanding African ethical approaches to suicide, assisted suicide, termination of pregnancy, surrogacy, organ donation and euthanasia. The ‘ubuntu-botho’ chapter also points out problems with the common practice in our clinics of using arbitrary language interpreters, thus disclosing confidential and potentially embarrassing health information to non-clinical staff members. An important point which emerges from the case studies in African ethics is the view that truth is not a pre-given entity but emerges from the conversation between the parties. Most African cities, and therefore secondary and tertiary healthcare institutions, serve urban populations whose parents or grandparents moved from rural areas with traditional, more communitarian practices. It would be interesting to know to what extent the ubuntu-botho approach is still applicable to this generation of Africans.

The ‘Ethics in nutrition: An African perspective’ chapter describes the interesting situation in Rwanda where the policy of ‘one cow per poor household’ is implemented by the state to prevent stunting and malnutrition by easy access to dairy products. However, this also carries risks: mothers with cows stop breastfeeding their babies earlier, thus denying the infants the beneficial effects of breastfeeding. This case study is an illustration of the difficulty in predicting unintended consequences of well-meaning health policies.

The many ethical issues faced by social workers in deprived areas are described. Social workers in South Africa are confronted with unusually high levels of sexual violence against women and children. The need to interact with offenders also raises ethical dilemmas for the social workers, especially in the context of mandatory reporting requirements. Sexual violence used as a weapon of war in African conflicts, such as in the Democratic Republic of the Congo, devastates communities. Social workers also experience a sense of betrayal, discomfort and ethical conflicts in their sense of responsibility to victims versus the need to assist in very necessary interventions to rehabilitate offenders. The high rates of violence in South Africa also mean that many social workers themselves are survivors of violence and sexual violence, which further compounds the distress and internal conflict affecting them.

The heavy workload exacerbates the research-practice gap in all sectors, with most research being carried out in the developed world. The importance of ‘cultural competence’ is highlighted in the physiotherapy chapters. This consists of self-awareness of personal culture, awareness of other cultures, sensitivity to other cultures and developing competence in diverse cultures. The authors point out that where there are high levels of illiteracy, patient autonomy is often compromised and medical paternalism is rife. A useful blueprint is provided in this chapter for developing cultural competence in physiotherapy in Africa. Inequality of access is a pervasive problem, and this, together with inadequate resources, compounds ethical dilemmas.

The nursing chapters recommend the inclusion of African philosophy in nursing training. The important point made is that current training emphasises individual autonomy while communities in which the nurses are situated are more comfortable with a communitarian approach, resulting in a clash of norms. Useful case studies in this and all chapters illustrate the need for Ubuntu in promoting empathy, respect and dignity. It is important from a treatment perspective that patients should not be fearful of admitting to using traditional medicines.

An important inclusion in the book is a section on curriculum guidelines for teaching healthcare ethics at undergraduate level. This chapter provides practical guidelines for developing an undergraduate medical ethics curriculum for healthcare workers. The framework consists of four learning units: ethics principles, ethical reasoning, professional skills and legal considerations. The chapter sets out the module content of the learning framework in units contained in useful tables. The substance of the learning units can be populated depending on the healthcare discipline, for example, nursing, occupational therapy and medical student training. The chapter also includes useful ideas for assessment.

This book will be useful for those setting up curricula and assessments for healthcare workers. Both healthcare workers and patients will benefit from an approach that is sensitive to the local context and takes into account a communitarian approach in addition to the usual principlist and utilitarian approaches to ethics taught in African healthcare institutions. The book provides important insights into ethical issues in the different healthcare disciplines on the African continent and the importance of locating ethical analysis in a local context understood by both healthcare worker and patient.

